# Electroencephalography reflects the activity of sub-cortical brain regions during approach-withdrawal behaviour while listening to music

**DOI:** 10.1038/s41598-019-45105-2

**Published:** 2019-07-01

**Authors:** Ian Daly, Duncan Williams, Faustina Hwang, Alexis Kirke, Eduardo R. Miranda, Slawomir J. Nasuto

**Affiliations:** 1 Brain-Computer Interfacing and Neural Engineering Laboratory, School of Computer Science and Electronic Engineering, University of Essex, Colchester, CO4 3SQ UK; 2 Digital Creativity Labs, Department of Computer Science, University of York, Heslington, YO10 5RG UK; 3 Interdisciplinary Centre for Computer Music Research, University of Plymouth, Plymouth, PL4 8AA UK; 4 Brain Embodiment Laboratory, Biomedical Sciences and Biomedical Engineering Division, School of Biological Sciences, University of Reading, Reading, RG6 6AY UK

**Keywords:** Emotion, Limbic system, Prefrontal cortex

## Abstract

The ability of music to evoke activity changes in the core brain structures that underlie the experience of emotion suggests that it has the potential to be used in therapies for emotion disorders. A large volume of research has identified a network of sub-cortical brain regions underlying music-induced emotions. Additionally, separate evidence from electroencephalography (EEG) studies suggests that prefrontal asymmetry in the EEG reflects the approach-withdrawal response to music-induced emotion. However, fMRI and EEG measure quite different brain processes and we do not have a detailed understanding of the functional relationships between them in relation to music-induced emotion. We employ a joint EEG – fMRI paradigm to explore how EEG-based neural correlates of the approach-withdrawal response to music reflect activity changes in the sub-cortical emotional response network. The neural correlates examined are asymmetry in the prefrontal EEG, and the degree of disorder in that asymmetry over time, as measured by entropy. Participants’ EEG and fMRI were recorded simultaneously while the participants listened to music that had been specifically generated to target the elicitation of a wide range of affective states. While listening to this music, participants also continuously reported their felt affective states. Here we report on co-variations in the dynamics of these self-reports, the EEG, and the sub-cortical brain activity. We find that a set of sub-cortical brain regions in the emotional response network exhibits activity that significantly relates to prefrontal EEG asymmetry. Specifically, EEG in the pre-frontal cortex reflects not only cortical activity, but also changes in activity in the amygdala, posterior temporal cortex, and cerebellum. We also find that, while the magnitude of the asymmetry reflects activity in parts of the limbic and paralimbic systems, the entropy of that asymmetry reflects activity in parts of the autonomic response network such as the auditory cortex. This suggests that asymmetry magnitude reflects affective responses to music, while asymmetry entropy reflects autonomic responses to music. Thus, we demonstrate that it is possible to infer activity in the limbic and paralimbic systems from pre-frontal EEG asymmetry. These results show how EEG can be used to measure and monitor changes in the limbic and paralimbic systems. Specifically, they suggest that EEG asymmetry acts as an indicator of sub-cortical changes in activity induced by music. This shows that EEG may be used as a measure of the effectiveness of music therapy to evoke changes in activity in the sub-cortical emotion response network. This is also the first time that the activity of sub-cortical regions, normally considered “invisible” to EEG, has been shown to be characterisable directly from EEG dynamics measured during music listening.

## Introduction

Music is an important part of the human experience for many people. It provides entertainment and social bonding, facilitates communication, and helps shape a sense of identity^1^. Music may be described as a form of emotional communication^2^ as it provides a way for people to communicate complex emotions without written or spoken language, both directly (via performance) and indirectly (via recording). This potential for music to evoke and communicate emotions makes it an important tool for investigating the neural correlates of emotions.

Music has been shown in numerous studies to change activity in core brain networks that underpin emotion^3–6^. Specifically, meta-analysis of functional magnetic resonance imaging (fMRI) studies examining music-induced emotions has shown significant changes in activity in a number of regions of the brain, including the amygdala, the hippocampal formation, the right ventral striatum, the left caudate nucleus, the pre-supplementary motor cortex, the cingulate cortex, and the auditory cortex^3^.

Although many of the same regions of this sub-cortical emotional response network are activated by a variety of reward stimuli, including music (see detailed review by Koelsch^3^), music also involves activity in the hippocampus^7^, a region of the brain not activated by other reward stimuli. This suggests that music-induced emotions are not just related to reward responses and thus it is important to study music as a unique type of stimulus.

Furthermore, music provides a unique tool to study affective responses to stimuli. Music is highly dynamic in its temporal structure, with parameters that evolve over timescales ranging from milliseconds to minutes^8^ and, consequently, music is able to produce dynamic and rapid changes in affective responses. Crucially, music is used in day to day life across cultures to communicate and modulate emotions^8,9^, and is therefore an ecologically relevant choice of stimulus.

A variety of neurological and psychiatric disorders, including pathological anxiety, depression, and schizophrenia, are characterized by structural and/or functional abnormalities in both the limbic and paralimbic systems^10,11^. These systems include the amygdala, hippocampus, cingulate gyrus, thalamus, and the cerebellum^3,12,13^. The large volume of fMRI-based evidence suggesting that music can change activity within these brain regions has encouraged investigation of the possible therapeutic effects of music in treating these diseases and conditions^3,10,11^.

A number of models of affect (the emotional response of an individual to a stimulus) have been proposed, including single and multidimensional classification models that attempt to identify discrete axes along which affective responses may be mapped. One of the most widely used models is the two-dimensional circumplex model of affect^14^. This model seeks to describe affect along two axes: valence (the positivity or negativity of the response) and arousal (the level of energy or intensity of the response). The scales of these two axes are assumed to be approximately linear, and individual affective states can be mapped onto the resulting two-dimensional space. For example, ‘fear’ may be described as negatively valenced (unpleasant) and highly arousing (intense), and would therefore be mapped onto the top left of the valence-arousal space.

Affective responses may be described in terms of approach-withdrawal response behaviour, which describes how an individual responds to joyful or fear-inducing stimuli^15^. Specifically, stimuli that evoke positive or negative emotions may produce an approach-withdrawal response^14^ (sometimes referred to as the fight or flight response^16^).

Music stimuli that produce approach-withdrawal responses have been shown, in numerous fMRI studies, to evoke activity within parts of the affective response network including the limbic and paralimbic systems, the superficial amygdala, mediodorsal thalamus, and the nucleus accumbens^3,4,17–20^. Together these brain regions constitute a network that modulates the approach-withdrawal response to music and to other socio-affective cues^3^.

Approach-withdrawal behaviour is also reflected in the electroencephalogram (EEG). A considerable body of evidence has shown that the relative valence induced by a stimuli in an individual affects the inter-hemispheric asymmetry within the prefrontal cortex^21–24^. This has led to the development of the “hemispheric valence hypothesis”^23,25^, which states that approach-related emotions are largely processed in the left frontal cortex and withdrawal-related emotions are largely processed within the right prefrontal cortex^26^. This hypothesis is supported by work by Heller, 1993, which suggests that the parietotemporal cortex in the right hemisphere is involved in autonomic and behavioural arousal^27^. This observation is further expanded upon in work by Rogenmoser and colleagues, which suggests that arousal is associated with suppression of alpha power in the right posterior cortex, and valence is associated with increases in theta power in the left frontal lobe^28^.

Motivated by these observed relationships between prefrontal asymmetry in the EEG and approach-withdrawal behavioural responses to music, EEG-based music therapy interventions have been developed which monitor prefrontal EEG asymmetry as an indicator of changes in music-induced emotions^29–32^.

However, EEG and fMRI measure very different brain processes and, in many cases, it is not clear how they relate to each other^33^. Specifically, the EEG measures summed electrical field potentials arising from the synchronized neural activity of millions of cortical neurons^34^. By contrast, fMRI measures changes in concentrations of oxy-haemoglobin throughout the brain^33^.

EEG provides a direct measure of neural activity with a very high time resolution that allows study of neural activity at a variety of time scales^35^. Specific frequency bands in the EEG have been shown to relate to a variety of neural processes^26^ and precise, time and phase-locked changes in activity within the EEG have been shown to reflect time-limited responses to stimuli and other neural processes^36^. However, it is generally thought to be impossible to measure activity of sub-cortical neurons directly using EEG due to the poor spatial resolution, coupled with the EEG’s inability to measure neural activity at depths below the cortical surface^37^.

By contrast, fMRI measures changes in concentrations of oxy-haemoglobin in all areas of the brain with a very high spatial resolution (Huster, Debener, Eichele, & Herrmann, 2012). However, the time-resolution of fMRI is much lower than EEG, with typical sample rates of around 0.5 Hz. Additionally, the haemodynamic response provides only an indirect measure of neural activity as it follows after neural activity with a delay of 2–4 s via the process of neurovascular coupling^38^. Thus, changes in fMRI activity are only functionally related to neural activity, and the nature of this functional relationship can be confounded by multiple factors, including repeated stimulus presentation, measurement noise, and imprecise understanding of neurovascular coupling^33,39,40^.

Consequently, it is not yet well understood how processes measured by EEG relate to processes measured by fMRI. Although some recent work has begun to explore relationships between the EEG and activity in the amygdala as measured by fMRI^41^, the relationships between EEG correlates of approach-withdrawal behaviour (the hemispheric valence hypothesis) and the wider sub-cortical network of brain regions involved in approach-withdrawal behaviour identified by fMRI, are not clear. Furthermore, music is a dynamic, socio-affective, ecologically relevant stimulus^8^, which evokes activity in a different affective response sub-network of brain regions than other affective stimuli^3^ and is, therefore, worth investigating as a unique stimulus type. Therefore, we set out to measure the relationships between music-induced changes in affect, EEG, and fMRI brain activity.

EEG is a nonlinear and nonstationary process^34^ and can only be treated as linear for short time periods after the presentation of phase-locked stimuli (e.g. event-related potentials^36^). Music is not a phase-locked stimulus and the neural correlates of music-induced changes in affect, most prominently the prefrontal asymmetry, are known to be nonlinear^42,43^. Therefore, to explore nonlinear responses to music in the EEG, and how these nonlinear responses reflect sub-cortical brain activity, we also use Shannon entropy^44,45^ to measure the disorder of the EEG asymmetry over time. The greater the entropy of the asymmetry, the greater the disorder of the observed asymmetry values, i.e. the less predictable the EEG asymmetry time series.

Combining EEG and fMRI measurement modalities has been shown to have added benefits in inferring the statistical relationships between these disparate processes^33,46^. We hypothesise that this combined modality recording may help shed new light on the functional relationships between EEG and fMRI during music listening.

In summary, music is an ecologically relevant stimulus that provides a unique and powerful tool for studying neural correlates of affect. It has numerous potential therapeutic applications that are based on the observed ability of music to modulate activity in the limbic and paralimbic systems. There are also a growing number of neural technology systems that use measurement of the prefrontal EEG asymmetry as a neural correlate of approach-withdrawal behaviour in the EEG. However, the functional relationship between prefrontal EEG asymmetry and the sub-cortical networks associated with approach-withdrawal behaviour is unclear.

We set out to investigate this functional relationship with the following hypotheses.

H1: For approach-withdrawal responses to music stimuli, prefrontal EEG asymmetry significantly relates to activity within parts of the sub-cortical emotion response network.

H2: More specifically, the EEG significantly relates to changes of activity within parts of the limbic system in response to music.

## Methods

### Participants

Recruitment was done via email advertisement. Participants were eligible for inclusion if they were aged between 20–30 years of age, right-handed, with normal or corrected to normal vision, with normal hearing, and if they did not have psychological or neurological health problems. Participants were also asked to complete a “Short test of musical preferences” (STOMP) questionnaire^47^ to identify the intensity of their likes and dislikes for different musical genres. Any participants who reported a score of 1 (strong dislike) to two or more genres, or a score of 1 for classical music were excluded at this stage. A strong dislike of classical music, in particular, was excluded due to the use of classical music in our experiments.

Participants were further screened to ensure they met the criteria for safe inclusion in joint EEG-fMRI experiments, as set out by the University of Reading MRI safety committee.

After screening, twenty-one (21) individuals participated in the study. Participants had a mean age of 24 (±2.6, range = 20–29). All participants were right-handed, and ten participants were female. Participants received £20.00 (GBP) for their participation in the study.

### Ethics

The study was reviewed according to the procedures of the University of Reading research ethics committee and given favourable opinion for conduct. All experiments were performed in accordance with all relevant guidelines and regulations. Informed written consent was obtained from all participants.

### Stimuli

Two sets of stimuli were used: generated music and classical music. Generated music stimuli were used to avoid changes in affective state resulting from familiarity due to previous exposure or repeated listening to a piece of music^48^ (generated music will have never been heard before by the participants), while classical music pieces were also used to investigate the stronger affective responses not possible with our generated music^49^. The purpose of this study was not to compare these music types, but to more thoroughly investigate neural correlates of affective responses to music using different music types.

#### Generated music

A number of music pieces were generated, which were designed to induce a range of key affective states uniformly distributed over the valence-arousal circumplex^14^. Specifically, a set of 36 musical excerpts was generated to target 9 different regions in the valence-arousal circumplex model of emotion (4 excerpts per region): high, neutral, and low valence combined with high, neutral, and low arousal. These regions were defined based on our earlier work, in which we showed that we were able to differentiate neural and physiological responses to generated music that targeted each of these regions of the valence-arousal circumplex^50,51^. Each excerpt was 40s long, and designed to target one region of the valence-arousal circumplex for the first 20s and then a different region for the next 20s. These durations were chosen to provide adequate time to experience changes in music-induced emotions^52^, while allowing for an experiment design that included sufficient numbers of trials for good statistical power.

The music generator used was an affectively-driven algorithmic composition system based on an artificial neural network. This was previously described and evaluated for its ability to induce targeted affect^53^ and has been validated in separate experiments with 22 participants self-reporting their affective states^54^ and with 20 participants undergoing physiological measurements while listening to generated music^55^.

#### Classical music

Four pieces of classical solo piano music were also used as stimuli, with the aim of covering most of the valence-arousal circumplex. Pieces were also chosen based on prior investigations of their effects on neural activity (to allow for comparative analysis) and prior investigations into their effects on a listener’s affective state^56^.

Specifically, the following four pieces of classical music were used as stimuli.

1. Prelude Opus 32, Number 5, by Sergei Rachmaninoff
2. Étude Opus 10, Number 3 in E major by Frédéric Chopin
3. Variations sérieuses Opus 54 (on a theme in D minor) by Felix Mendelssohn
4. Piano Sonata number 4, Opus 7, in E-flat major (the Grand Sonata) by Ludwig van Beethoven

Rachmaninoff’s prelude has been previously investigated in an EEG study on the effects of classical music on the brain and related changes in tension^56^. The authors reported that the piece induced significant changes in tension in all listeners. Based on this study, and our own experience, this piece may be described as inducing calm emotions with a high valence throughout, and inducing time-varying changes in arousal, ranging from low to medium/high levels.

Chopin’s Étude was previously investigated in an fMRI study looking at correlates of music-induced changes in arousal^8^. The authors compared the piece to synthetic music and reported significant time-varying changes in arousal. This piece may be described as inducing sad emotions with low valence and inducing low levels of arousal at the start, changing to high arousal as the piece progresses.

Mendelssohn’s Variations sérieuses was previously investigated in an fMRI study exploring the temporal dynamics of music-induced valence and arousal^57^. The authors reported a steady, induced neutral valence and changing levels of arousal reported by listeners as the piece progresses.

Finally, Beethoven’s sonata was chosen to complete the range of affective states covered by the stimuli. Specifically, we judged this piece to be likely to produce high valence and high arousal in listeners, while being comparable in structure to the other pieces. Beethoven’s piano sonatas have been the subject of considerable evaluation of their contribution to emotional responses^58^.

The classical music pieces had different durations (see Table 1).

**Table 1:** Classical music pieces used as stimuli in our experiment.

Piece	Composer	Performer	Targeted affective state	Duration (s)
Prelude Opus 32, Number 5	Sergei Rachmaninoff	Sergei Rachmaninoff	High valence, low arousal changing to high arousal	127
Étude Opus 10, Number 3 in E major	Frédéric Chopin	Murray Perahia	Low valence, Low arousal changing to high arousal	150
Variations sérieuses Opus 54 (on a theme in D minor)	Felix Mendelssohn	Benjamin Frith	Neutral valence, Changing arousal from low to high and back to low	170
Piano Sonata number 4, Opus 7, in E-flat major (the Grand Sonata)	Ludwig van Beethoven	Dieter Zechlin	Low valence changing to high valence, neutral arousal	158

### Paradigm

The experiment session comprised two stages. In the first stage (approximate duration: 30 minutes), participants were asked to listen to the generated music excerpts and report their felt affective states on the valence-arousal circumplex. Reporting was performed via the FEELTRACE interface, a joystick-controlled interface, which allowed participants to report their current affective states by moving a cursor in the two-dimensional valence-arousal space. This method for reporting affect has previously been validated with 24 participants^59^. In the second stage (approximate duration: 30 minutes), participants were asked to listen to the classical music pieces and report their felt affective states. The session with generated music was completed first to avoid biasing participants’ responses. Specifically, we are most interested in the subtle differences in affect arising from different pieces of generated music, not the large differences that may result from comparing generated music with classical music.

Participants performed three types of trial for each piece of music.

1. Music only trials: music was played to the participants, who were asked to look at a fixation cross for the duration of the trial without moving and while just listening to the music.
2. Music and reporting trials: participants listened to music and simultaneously used an MRI-compatible joystick (Mag Design and Engineering, UK) to report their affective states via FEELTRACE.
3. Reporting only trials: participants were presented with a recording of their FEELTRACE movements recorded during a previous trial. They were asked to track this movement with the joystick i.e. to visually track a recording of this movement and, simultaneously control the cursor to follow this movement. No music was played during this trial. Thus, this trial acted as a movement and visual attention control; participants would produce approximately the same FEELTRACE-related movements (including eye movements) as in the ‘music and reporting trials’, while not listening to music and thus not experiencing specific music-induced emotions.

Within a single trial participants were presented with a fixation cross for a minimum of 1 s. The task then began upon the next transistor-transistor logic (TTL) trigger from the MRI machine with a random offset uniformly drawn from between 0–2 s. As the MRI was acquired with a repetition time (TR) of 2 s, the fixation cross thus remained on screen for between 1–3 s. The task then took 40 s to complete and comprised one of the three trial types described above. This was followed by a 0.5 s break before the next trial.

**Figure 1:**
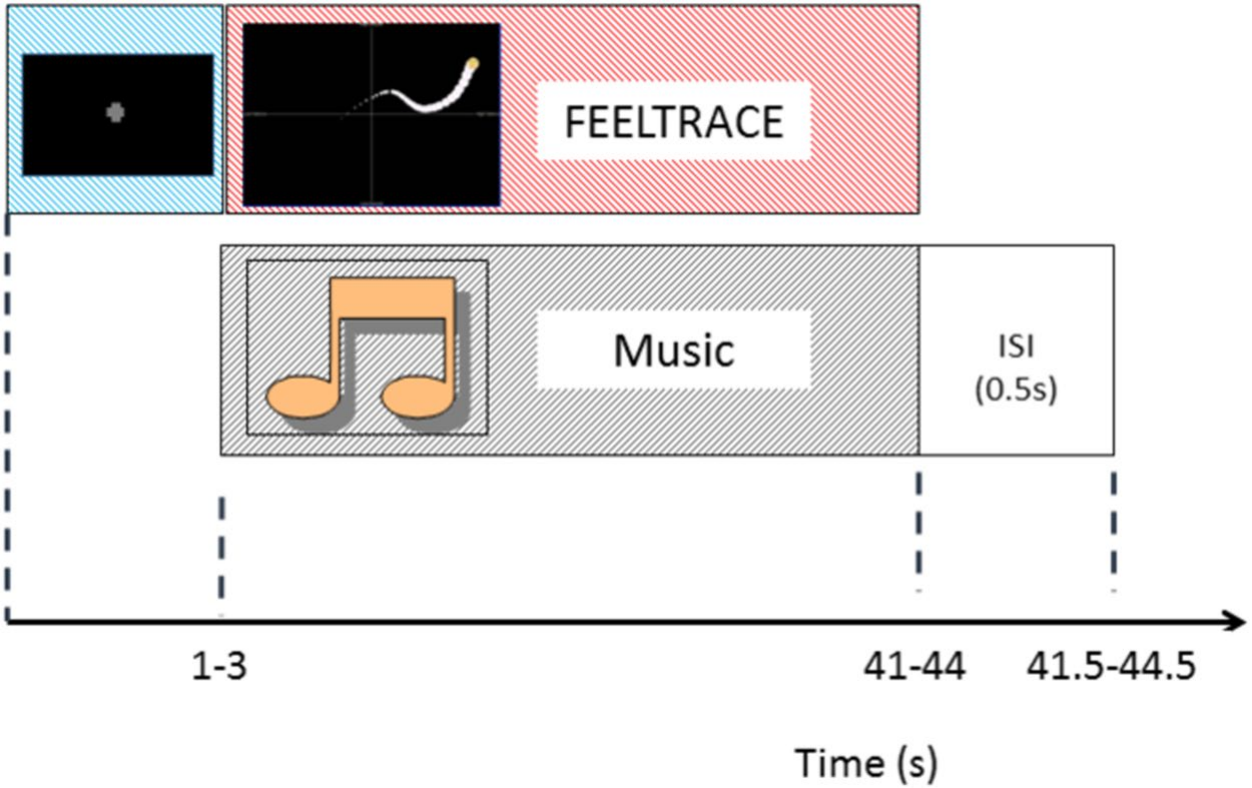
Timing of events within a single trial.

The timing of a single trial of the experiment is illustrated in Fig. 1.

All audio stimuli were played via MRI-compatible headphones (NordicNeurolab, Norway), which participants wore throughout the experiment. Participants also wore ear-plugs to protect their hearing from the acoustic noise generated by the scanner. Before the experiments began we adjusted the music volume to a level that was comfortable and allowed each participant to hear the music above the noise of the scanner.

The order of presentation of the 36 trials was pseudo-randomized, with a constraint that each ‘reporting only’ trial was always preceded at some point in the experiment session by a corresponding ‘music and reporting’ trial for the same piece of music.

The ‘music only’ trial was used to distinguish brain activity related to music-induced changes in affective state from brain activity related to using the FEELTRACE interface. Specifically, participants were assumed to respond affectively to music in the same way regardless of whether they were using FEELTRACE or not.

To achieve this, participants’ FEELTRACE reports from their ‘music and reporting trials’ were copied to their ‘music only trials’. Brain regions that significantly co-vary with reports of affect during the ‘music only trials’ and during the ‘music and reporting trials’, but not during the ‘reporting only’ trials are, thus, highly likely to be the regions that are involved in affective responses to music and not involved in control of FEELTRACE.

The session involving generated music was split into three runs of approximately 10 minutes each, with a 1-minute break between runs. Each run contained 12 trials in pseudo-random order.

In the session involving classical music, all 4 music pieces were played in pseudo-random order in the same run using the three trial types described above.

Thus, the classical music run was approximately 30 minutes long. The participants were asked to complete the generated music runs first and then asked to complete the classical music run. The response techniques were the same for both the generated and the classical music stimuli types.

In between the generated music stage and the classical music stage of the experiment, participants were asked to complete a two minute 2-back audio-based memory listening task. This served as a ‘washout’ task to reduce the likelihood of participants evaluating the classical music in comparison to the generated music. The 2-back task consisted of 5 farm-yard animal sounds, which were played to participants in random order (200 ms per sound with a 200 ms inter-stimulus interval). Participants were asked to press a button on the joystick when they heard a sound that had been previously heard 2 steps back.

### Recording

EEG was recorded via a 32 channel (31 channel EEG and 1 channel electrocardiogram) MRI-compatible BrainAmp MR and BrainCap MR EEG system (Brain Products Inc., Germany). EEG was recorded at 5,000 Hz, without filtering (an analogous approach to^60^), and with an amplitude resolution of 0.5uV. The reference electrode was placed at FCz. All electrodes were placed according to the International 10/20 system. Impedances were kept below 15kΩ throughout the experiments.

The MRI was recorded using a 3.0 Tesla Siemens Magnetom Trio scanner with Syngo software (version MR B17) and a 37-channel head coil. The set of scanning sequences used was composed of a gradient echo planar localizer sequence, followed by an anatomical sequence (field of view = 256 × 256 × 176 voxels, TR = 2020 ms, TE = 2.9 ms, voxel dimensions = 0.9766 × 0.9766 × 1 mm, flip angle = 9 degrees), and then a set of gradient echo planar functional sequences. Finally, a gradient echo planar field mapping sequence was applied.

Functional sequences were recorded with a repetition time of 2000 ms, echo time of 30 ms, field of view of 64 × 64 × 37 voxels, voxel dimensions of 3 × 3 × 3.75 mm, and flip angle of 90 degrees.

Co-registration of EEG and MRI scans was performed via BrainVision recording software (BrainProducts, Germany). Stimuli were presented via custom written software based on Psychtoolbox^61^.

### Pre-processing

#### EEG

The imaging artefact was first attenuated using the Average Artefact Subtraction (AAS) method^62^, as implemented in Vision Analyzer software (BrainProducts). The ballisto-cardiogram artefact was also removed from the EEG via the AAS method. The cleaned EEG was then visually checked to confirm successful attenuation of the artefacts.

#### fMRI

Functional magnetic resonance imaging (fMRI) images were pre-processed using SPM12^63^ software running within Matlab 2014b (Mathworks, USA). Slice-time correction was first applied using the first slice of each run as a reference image. Movement-related artefacts were then removed from the images via the realignment and unwarping approach proposed by Friston *et al*.^64^. Field maps, recorded from each participant, were used to remove movement artefacts from the images and to correct for participant-specific image warping effects. A separation of 4 mm was used with a Gaussian smoothing kernel of 5 mm, and a 2nd degree spline interpolation was used for the realignment, followed by a 4th degree spline interpolation function for the unwarping.

Co-registration was then applied to register the functional images against high-resolution anatomical images for each participant. A 7 mm Gaussian smoothing kernel was used with a 4th degree spline interpolation function.

Images were then normalized to a T1 template image with a 4th degree spline interpolation. Finally, functional images were smoothed via a Gaussian filter with a full-width at half maximum of 8 mm on each dimension.

### Analysis and statistics

#### FEELTRACE reports

FEELTRACE reports were first visually inspected to confirm that participants were able to use the interface correctly to report along both axes (valence and arousal). The felt affects reported by participants via FEELTRACE were then compared to the affective states targeted by each piece of music via a correlation analysis.

#### fMRI

Changes in hemodynamic blood flow related to changes in valence and arousal were investigated. Statistical parametric mapping was used to identify voxels that significantly co-varied with participant’s reports of their felt valence and arousal.

First, for each stimulus (generated and classical music) and for each dimension of the affective space (valence and arousal), voxels were identified that significantly co-varied with reports of felt affective states during all the music listening trials (that is, both the ‘music and reporting trials’ and the ‘music only trials’). The family-wise error rate was used (corrected p < 0.05), to correct for multiple comparisons.

Second, voxels that significantly co-varied with participant reports during ‘reporting only trials’ were identified. These voxels indicate brain regions related to control of FEELTRACE but not to the act of listening to the music and the associated experience of music-induced emotions.

Finally, a contrast was used to identify voxels that significantly co-varied with reports of affect while participants listened to music but not while they used FEELTRACE in the absence of music.

Specifically, two sets of voxels were identified: set A contained voxels that significantly co-varied with participants’ reports of their emotions during the ‘music only’ and the ‘music and reporting’ trials, and set B contained voxels that significantly co-varied with control of the FEELTRACE interface but not with music-induced emotions, i.e. voxels that significantly co-varied with FEELTRACE reports during the ‘reporting only’ trials. We then identified a new set, C, as the set difference of A and B via

$$C=A\backslash B.$$

Therefore, set C contained all the voxels that co-varied with FEELTRACE reports during music listening but that did not co-vary with use of FEELTRACE alone (i.e. motor control of the joystick and visual tracking of the display screen).

The significance level of the exclusion contrast was set at p < 0.05. This revealed voxels that significantly co-varied with music-induced changes in affective states but that were not related to the use of the reporting mechanism (e.g. voxels related to visual processing or motor control of the arm to move the joystick while using FEELTRACE).

In all analysis, independent variables were convolved with a canonical hemodynamic response function (SPM). Additionally, movement trajectories were treated as confounds in the general linear model (GLM).

#### EEG

Pre-frontal EEG asymmetry was investigated as an indicator of music-induced changes in affective states. EEG was first re-referenced using a common averaged reference (CAR) scheme. Independent component analysis was then used to identify and remove any remaining artefacts from the signal that persisted after previous pre-processing stages. Specifically, second order blind source separation (SOBI)^65^ was used to identify a de-mixing matrix which maximised the statistical separation between the independent components (ICs). ICs were then visually inspected by an experienced EEG analyst (author ID, 7 years’ experience), who was blinded to information about the EEG epochs (FEELTRACE reports and which piece of music the participant listened to) to minimize potential bias. Independent components that were judged to contain artefacts were identified and removed from each participant’s data. The remaining components were then recombined, resulting in cleaned EEG.

On average 8.9 ICs were removed from each participant’s EEG data (STD. = 2.6). Visual inspection of the cleaned EEG then verified that the artefacts had been correctly removed.

Asymmetry features were then extracted following the method defined in^66^. Specifically, EEG was band-pass filtered in the range 1 to 45 Hz via a third order Butterworth filter, and time series’ of band-power activity were extracted within four key frequency bands of interest, the delta (0–4 Hz), theta (4–8 Hz), alpha (8–12 Hz), and the beta (13 to 20 Hz) frequency bands. These frequency bands were selected due to their relationships with affective responses and other cognitive processes, previously reported in the literature^21,26,28,50^. Laplacian derivations were extracted, as a set of time-series, from the right and left pre-frontal cortex centred on channels F3 and F4, with reference channels FP1/2, F7/8, Fz, and C3/4. A time-series of asymmetry values was then calculated as the difference between left and right pre-frontal cortex Laplacian derivations.

#### EEG-informed fMRI

The relationship between pre-frontal EEG asymmetry and the fMRI activity recorded from the participants was investigated by adopting an EEG-informed fMRI analysis approach^33^. Specifically, a time series of EEG prefrontal asymmetry values was constructed for each frequency band for which a significant difference in pre-frontal asymmetry was observed from the EEG analysis, co-registered to the start time of the first fMRI sample point, and down-sampled to 0.5 Hz (equivalent to the sample rate of the MRI scanner, TR = 2 s).

These time series of pre-frontal asymmetry values were used as explanatory variables in the GLM (SPM12). Specifically, an F-contrast was constructed for each participant to identify voxels that significantly co-varied with asymmetry (family wise error rate (FWE) corrected, p < 0.01).

#### EEG dynamics informed fMRI

EEG is highly dynamic and non-stationary^67^. EEG dynamics have been observed to fluctuate in relation to affect^68^ and EEG asymmetry is also known to be highly dynamic, even when participants attend to stimuli that aim to induce a single affective state^69^.

Therefore, we explored the dynamics of music-induced changes in prefrontal asymmetry in the EEG and how these dynamics relate to hemodynamic activity in sub-cortical brain areas. We sought to understand how rapid changes in EEG relate to slower BOLD changes elsewhere in the brain and how information at different spatial and temporal scales is integrated during the experience of affect.

There are multiple methods for investigating the temporal dynamics of EEG processes, including measures of autocorrelation, Markov models, and measures from information theory such as entropy^43,70^. In this study, we characterized ongoing fluctuations of the Shannon entropy, a metric from information theory. This is motivated by previous observations that changes in affect can change the Shannon entropy of the EEG^44,45,68^.

Shannon entropy is defined as $H(X)={\sum }_{x}P(x){\mathrm{log}}_{2}[P(x)]\,$, where P(x) denotes the probability that signal X is in state x and P log_2_(P) is defined to be 0 if P = 0.

We first measured the Shannon entropy of the prefrontal asymmetry of the EEG that was recorded during each music-listening trial, in each of the frequency bands for which a significant difference in pre-frontal asymmetry was observed from the EEG analysis. We then compared Shannon entropy between high and low valence trials to verify whether the Shannon entropy of EEG asymmetry could be used to differentiate high versus low valence affective states during music listening. This was motivated by a desire to compare how the information content of the EEG co-varies with affect.

We went on to explore the relationship between the entropy of the EEG asymmetry and BOLD activations via an EEG-informed fMRI analysis. Specifically, we extracted a time series of Shannon entropy values over all music listening trials. We then used this entropy time series as an independent variable in a GLM to identify brain regions, from the fMRI recording, that significantly co-varied with changes in the dynamics of the EEG asymmetry.

## Results

### Participant reports

**Figure 2:**
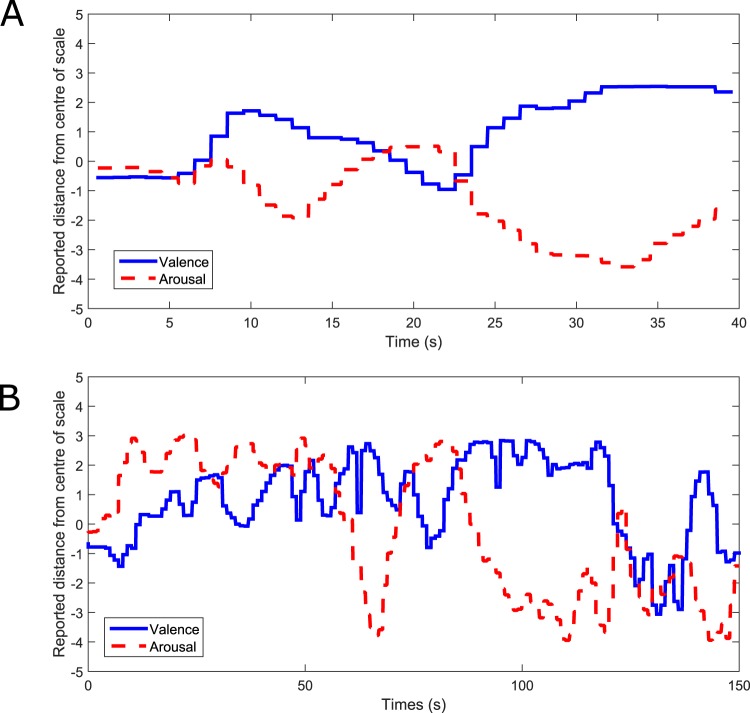
(**A**) An example of a participant’s FEELTRACE report during one 40 s music listening and reporting trial, while the participant listened to generated music. (**B**) An example of a participant’s FEELTRACE report while listening to a piece of classical music (Étude Opus 10, Number 3 in E major by Frédéric Chopin). The y-axis indicates the distance from the ‘neutral’ position of the participant’s reports of their felt valence and arousal (e.g. positive values indicate positive valence/arousal).

Visual inspection of participants’ reports of their affective states (valence and arousal, via FEELTRACE) while listening to the generated music revealed large variability. Participants did not consistently report a single state, but rather an ever-changing trajectory of affective states over time. An example of this is illustrated in Fig. 2(A).

Note that participant reports of their felt affective states over the 40 s music listening period are highly dynamic. There is a clear change in state shortly after 20 s (the time when the music generator sought to change affective state), but there are also many smaller localized changes in affective states at multiple time points, suggesting a highly dynamic affective state trajectory induced by both the generated music stimuli and the classical music stimuli.

To confirm the effectiveness of the generated music at inducing the targeted affective states, the felt affective states reported by our participants were compared to the targeted affective states for each piece of generated music. Specifically, the FEELTRACE reports from all participants were separated into 20 s sub-trials, with each sub-trial encapsulating a reporting period during which the music generator was targeting a discrete affective state. We then measured the degree of correlation between the mean affective states reported by participants during each of these sub-trials and the affective states targeted by the music generator.

There were small, but significant, correlations between targeted and reported affective states over all participants on both the valence and arousal axes. Specifically, valence targeted by the generated music was significantly correlated with reported valence (r = 0.300, p = 0.013), as was also the case for targeted and reported arousal (r = 0.193, p = 0.045).

However, as these correlations between targeted and reported states are quite low, it is reasonable to conclude that participants often reported experiencing different affective states to those targeted by the generated music.

The participant’s reports of their affective states, while they listened to classical music, were also visually inspected and compared to previous literature in order to check if the music pieces were inducing a range of affective states and if these were similar to those reported in other studies. An example of participants’ reports of their valence and arousal while they listened to Étude Opus 10, Number 3 in E major by Frédéric Chopin is illustrated in Fig. 2(B). It may be noted that valence and arousal both change considerably as the music is played.

Interestingly, the arousal reported by our participants (and illustrated in Fig. 2(B)) can be visually compared to results reported in^8^, in which participants were asked to report their felt arousal on a continuous basis while listening to this same piece of music. A visually similar pattern of changes in arousal may be observed, suggesting a consistency in affective responses to this music over different groups of participants.

Taken together with the low correlation between targeted and reported affects during generated music listening, these results suggest that participants’ reports of their affective states while listening to music can be used as the independent variable (or ground truth measure) of their felt affect. It also suggests that it would be better to consider reported affect as a continuous variable, rather than a discrete one. We adopt this approach.

### fMRI

The fMRI recordings were first analysed individually to identify the sub-cortical affective response network.

#### Generated music

Voxels that co-varied significantly with valence or arousal, while participants listened to generated music, were identified by fitting a GLM to relate FEELTRACE reports (the dependent variable) to BOLD (the independent variables).

Voxels were identified that significantly co-varied with the FEELTRACE reports during the non-music (‘reporting only’) trials. These voxels indicate which brain regions exhibit activity related to using FEELTRACE (moving a joystick and following a cursor).

Finally, for each condition, contrasts were constructed to identify voxels that co-varied with felt affect while listening to music, but that did not co-vary with their reported affective states when no music was playing.

While participants were listening to generated music the brain regions that were found to co-vary significantly with valence (family-wise error corrected, p < 0.05) include the amygdala, auditory cortex, parts of the cerebellum, cingulate gyrus, right motor cortex, right occipital temporal cortex, and posterior temporal cortex.

**Figure 3:**
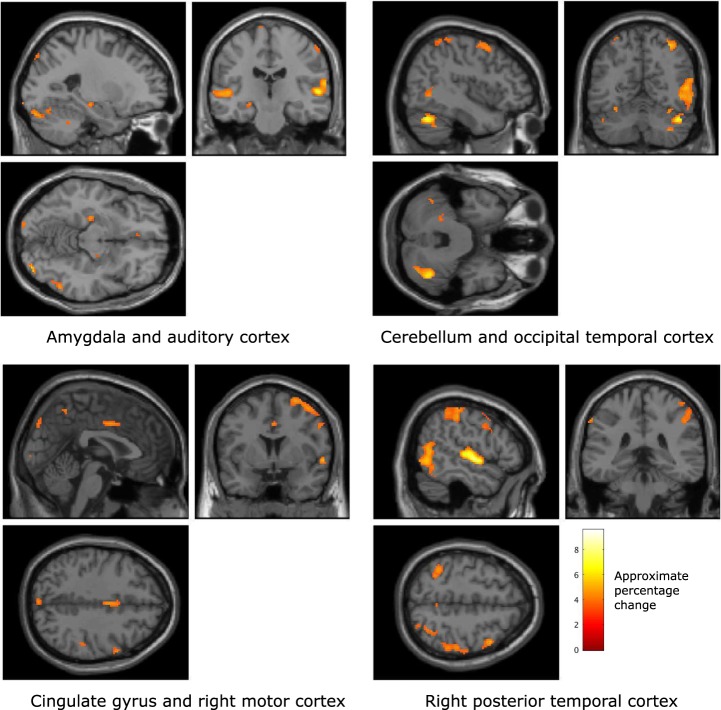
Brain regions that were found, over all participants, to co-vary significantly with reported valence during the generated music listening trials (p < 0.01, family wise error rate corrected). The colour scale indicates the approximate percentage change in BOLD activity in voxels where there is a significant change, as defined by SPM, between conditions.

Figure 3 illustrates the brain regions that were found to co-vary significantly with reported valence, but that did not co-vary significantly with use of the FEELTRACE interface, during the generated music trials.

Table 2 lists the local maxima BOLD associated with changes in valence during the generated music listening trials.

**Table 2:** Local maxima of regions with significant co-variation with reported felt affect, but not with movement, during generated music listening tasks (voxel size: 2 × 2 × 2 mm).

Affective dimension	Anatomical location	Voxels	MNI coordinates (mm)	p (FWE corrected, voxel level)	F score
Valence	Amygdala, right hemisphere	17	20, −10, −14	0.018	3.93
Valence	Amygdala, left hemisphere	22	−24, −20, −12	<0.001	4.35
Valence	Auditory cortex, right hemisphere	869	58, −18, 2	<0.001	9.22
Valence	Auditory cortex, left hemisphere	350	−56, −18, 2	<0.001	6.53
Valence	Cingulate gyrus	68	2, 0, 40	<0.001	4.34
Valence	Primary motor cortex, right hemisphere	663	24, −2, 68	<0.001	5.67
Valence	Posterior temporal cortex, right hemisphere	917	32, −60, 58	<0.001	5.03
Valence	Posterior temporal cortex, left hemisphere	295	−34, −54, 56	<0.001	4.62
Valence	Occipital temporal cortex	1141	44, −62, −30	<0.001	6.73
Valence	Cerebellum, left hemisphere	385	−32, −66, −18	<0.001	5.72
Arousal	Amygdala, right hemisphere	80	20, −14, −14	<0.001	6.08
Arousal	Amygdala, left hemisphere	9	−24, −20, −10	<0.001	4.68
Arousal	Auditory cortex, right hemisphere	1758	58, −14, 6	<0.001	9.57
Arousal	Auditory cortex, left hemisphere	1282	−60, −26, 8	<0.001	7.71
Arousal	Cingulate gyrus	160	0, 12, 38	<0.001	5.78
Arousal	Lateral orbitofrontal gyrus	102	−4, 34, −14	<0.001	5.69
Arousal	Lingual gyrus	168	−4, −48, 14	<0.001	4.93
Arousal	Parietal cortex	3724	44, −60, −30	<0.001	8.68
Arousal	Cerebellum, left hemisphere	585	−32, −64, −18	<0.001	4.95

BOLD activity recorded from voxels in the auditory cortex and the posterior temporal cortex were found to exhibit significant co-variations with participants’ reports of their felt valence. Additionally, voxels within the amygdala, the cingulate, the right hemisphere motor cortex, and the cerebellum were also observed to co-vary significantly with participants’ reports of their felt valence.

**Figure 4:**
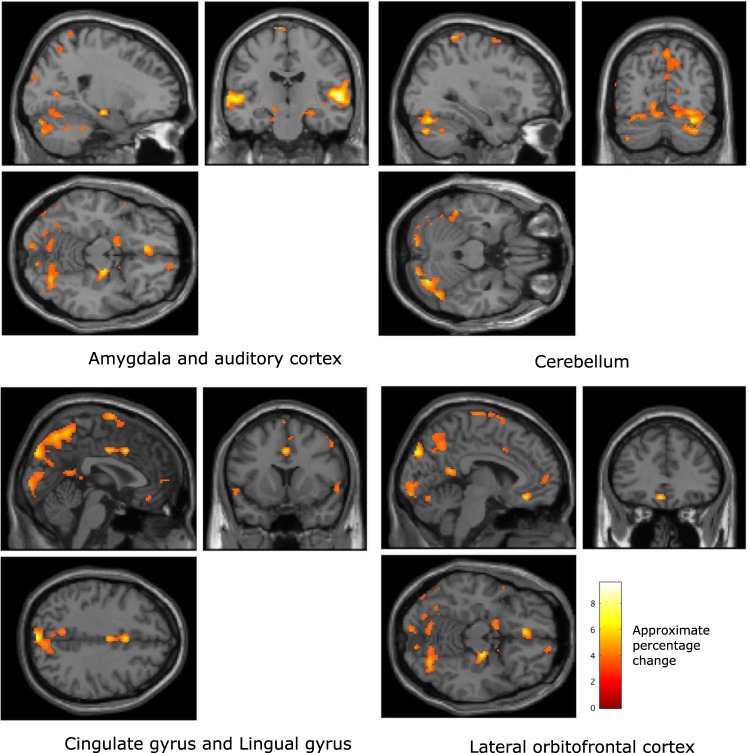
Brain regions that were found, over all participants, to co-vary significantly, with participants’ reports of their felt arousal during the generated music listening trials (p < 0.01, family wise error rate corrected). The colour scale indicates the approximate percentage change in BOLD activity in voxels where there is a significant change, as defined by SPM, between conditions.

Figure 4 illustrates the brain regions that co-varied significantly with participants’ reports of their felt arousal while listening to the generated music, and that were not found to be associated with the control of FEELTRACE

Table 2 lists the locations, sizes, and significance of the voxel clusters found to co-vary significantly with participants’ reports of their felt arousal during the generated music listening trials.

It may be noted that the amygdala, auditory cortices, and cerebellum all exhibited significant relationships with valence and arousal. However, the lingual gyrus, lateral orbitofrontal gyrus, and parietal cortex were only observed to relate to reported arousal, while the occipital temporal cortex only related to valence during generated music listening.

#### Classical music

**Figure 5:**
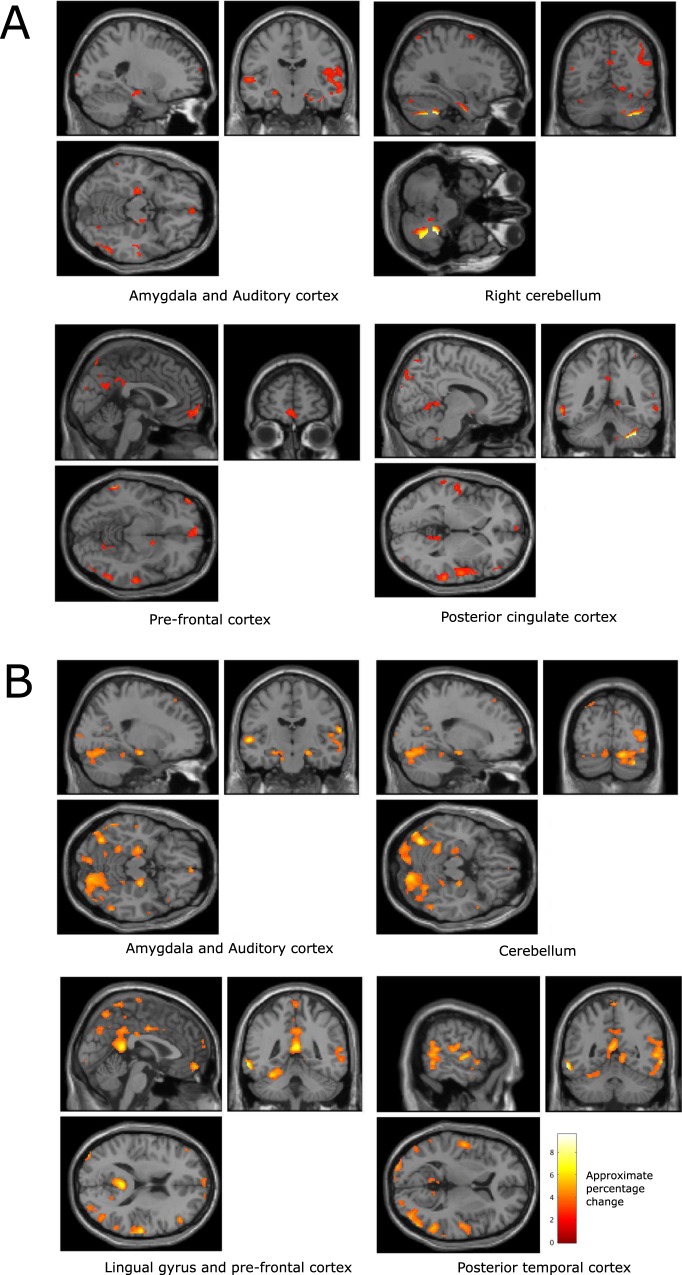
(**A**) Brain regions that were found, over all participants, to co-vary significantly with reported valence during the classical music listening tasks (p < 0.01, family wise error rate corrected). (**B**) Brain regions that were found to co-vary significantly with reported arousal during the classical music listening tasks (p < 0.01, family wise error rate corrected). The colour scale indicates the approximate percentage change in BOLD activity in voxels where there is a significant change, as defined by SPM, between conditions.

To verify that the results were not an artefact of the music generation process, the analysis was repeated for the classical music. The regions of the brain that significantly related to valence (family-wise error corrected, p < 0.01) were observed to include the amygdala, auditory cortex, and cerebellum (see Fig. 5(A) and Table 3). This activity significantly co-varied with participants’ reports of their valence, a result that corresponds to the brain regions found to co-vary with valence during the generated music listening tasks. Additionally, BOLD activity in the prefrontal cortex and the posterior cingulate cortex were also observed to significantly co-vary with felt valence during the classical music listening trials.

**Table 3:** Local maxima of regions with significant co-variation with reported affect, but not with movement, during classical music listening tasks (voxel size: 2 × 2 × 2 mm).

Affective dimension	Anatomical location	Voxels	MNI coordinates (mm)	p (FWE corrected, voxel level)	F score
Valence	Amygdala, right hemisphere	57	26, −14, −20	<0.001	4.96
Valence	Amygdala, left hemisphere	52	−24, −18, −14	<0.001	4.87
Valence	Auditory cortex, right hemisphere	1763	46, 4, −36	<0.001	10.11
Valence	Pre-frontal cortex	192	−2, 64, −8	<0.001	5.60
Valence	Posterior cingulate cortex, right hemisphere	174	12, −44, 2	<0.001	4.25
Valence	Cerebellum	350	38, −46, −40	<0.001	15.20
Arousal	Amygdala, right hemisphere	73	22, −14, −16	<0.001	5.57
Arousal	Amygdala, left hemisphere	106	−24, −16, −16	<0.001	4.80
Arousal	Auditory cortex, right hemisphere	1684	64, −18, 16	<0.001	6.07
Arousal	Pre-frontal cortex	102	4, 56, −12	<0.001	5.44
Arousal	Posterior cingulate cortex	1789	2, −42, 14	<0.001	6.83
Arousal	Cerebellum	1470	34, −78, −30	<0.001	6.02

Brain regions that exhibited BOLD activity that significantly co-varied with felt arousal during the classical music listening trials are illustrated in Fig. 5(B).

The results illustrate that similar patterns of BOLD activation were found to co-vary with reported arousal while participants listened to classical music, compared to those observed while participants listened to the generated music (see Table 3). However, the region of significant BOLD co-variation with reported arousal in the lingual gyrus was considerably larger during the classical music listening task, and additional regions of significant co-variation of the BOLD signal were also observed in the posterior temporal cortex.

### EEG

The relative differences in EEG asymmetry between high and low valence conditions, while listening to the generated music and classical music, were investigated in the delta, theta, alpha, and beta bands. The results of Kolmogorov-Smirnov (*ks*)-tests comparing asymmetry values between high and low valence conditions for each band revealed significant differences (p < 0.05, corrected for multiple comparisons via Holm-Bonferroni correction) in asymmetry between high and low valence in the alpha and beta frequency bands.

**Figure 6:**
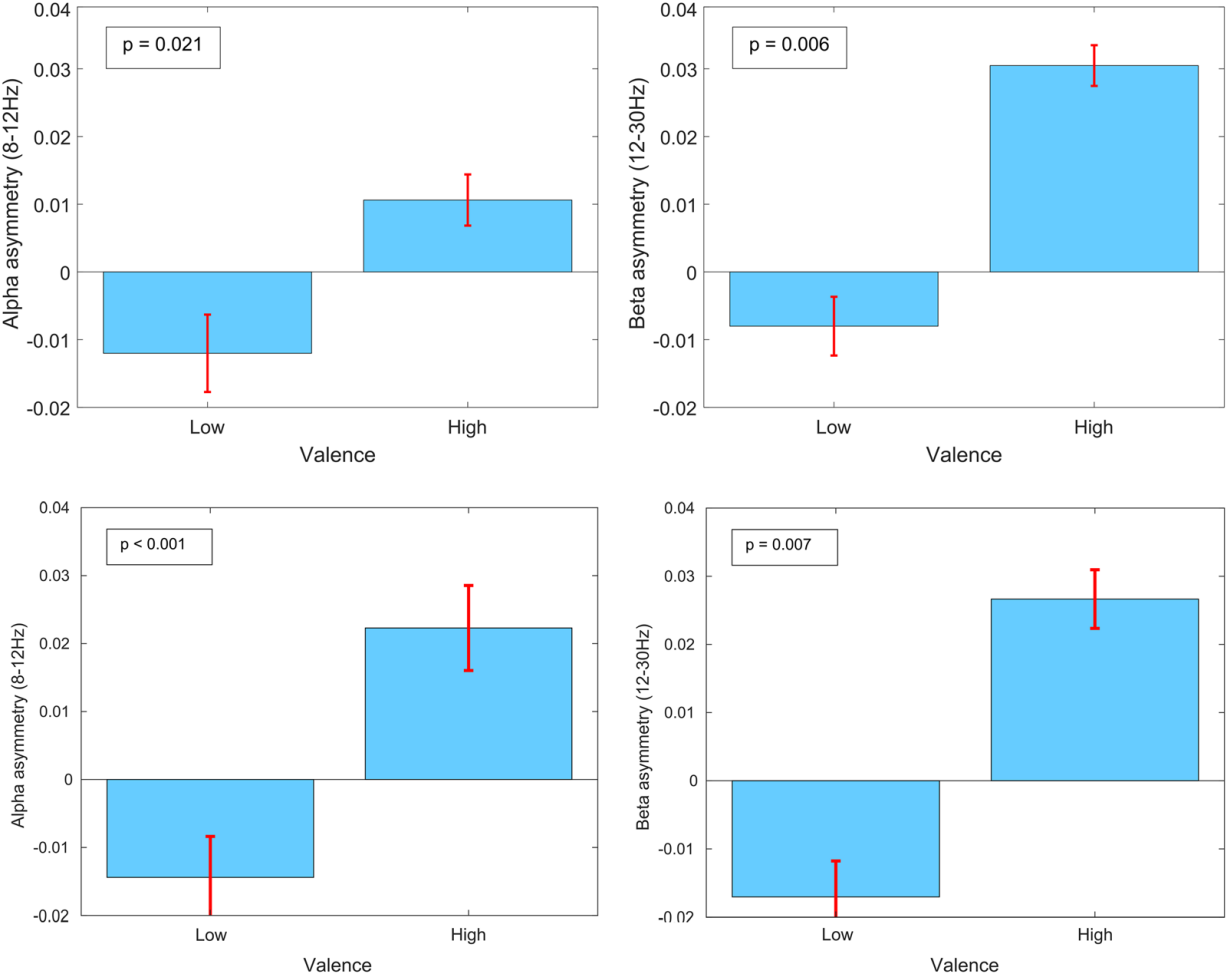
Mean and 95% confidence intervals of EEG asymmetry (left prefrontal cortex EEG activity minus the right prefrontal cortex EEG activity, measured in uV) in the alpha and beta frequency bands from all participants, while listening to the generated music (top row) and the classical music (bottom row). The significance level of the difference in asymmetry values between conditions is listed in the boxes on each figure.

The mean relative differences in EEG asymmetry in the alpha and beta bands between high and low valence, along with the associated statistical significance test results, are illustrated in Fig. 6.

It may be observed that EEG asymmetry in both bands was significantly larger when participants reported high valence compared to when they reported low valence.

Similar results were found for both generated and classical music listening, with significantly higher alpha and beta asymmetry observed when participants reported positive valence (p < 0.01, paired *ks*-test, corrected for multiple comparisons via Holm-Bonferroni correction).

As a control test we also evaluated the difference in asymmetry between high and low valence conditions during the ‘reporting only’ trials. In these trials participants did not hear any music, they just followed a previously reported FEELTRACE record visually and with the joystick. Thus no asymmetry should be found in these trials. Indeed, we did not find significant differences in asymmetry in these control trials in any frequency band (p > 0.05), confirming that differences in asymmetry relate to felt affect, not movement or visual attention. This control task also provides additional evidence to highlight that our observations are not a result of artefact contamination by the ballisto-cardiogram (BCG) artefact. Identical BCG removal was applied to both music listening and reporting only trials, while the only difference between these trials was whether music was played to participants, and hence whether they experienced changes in music-induced affect.

**Figure 7:**
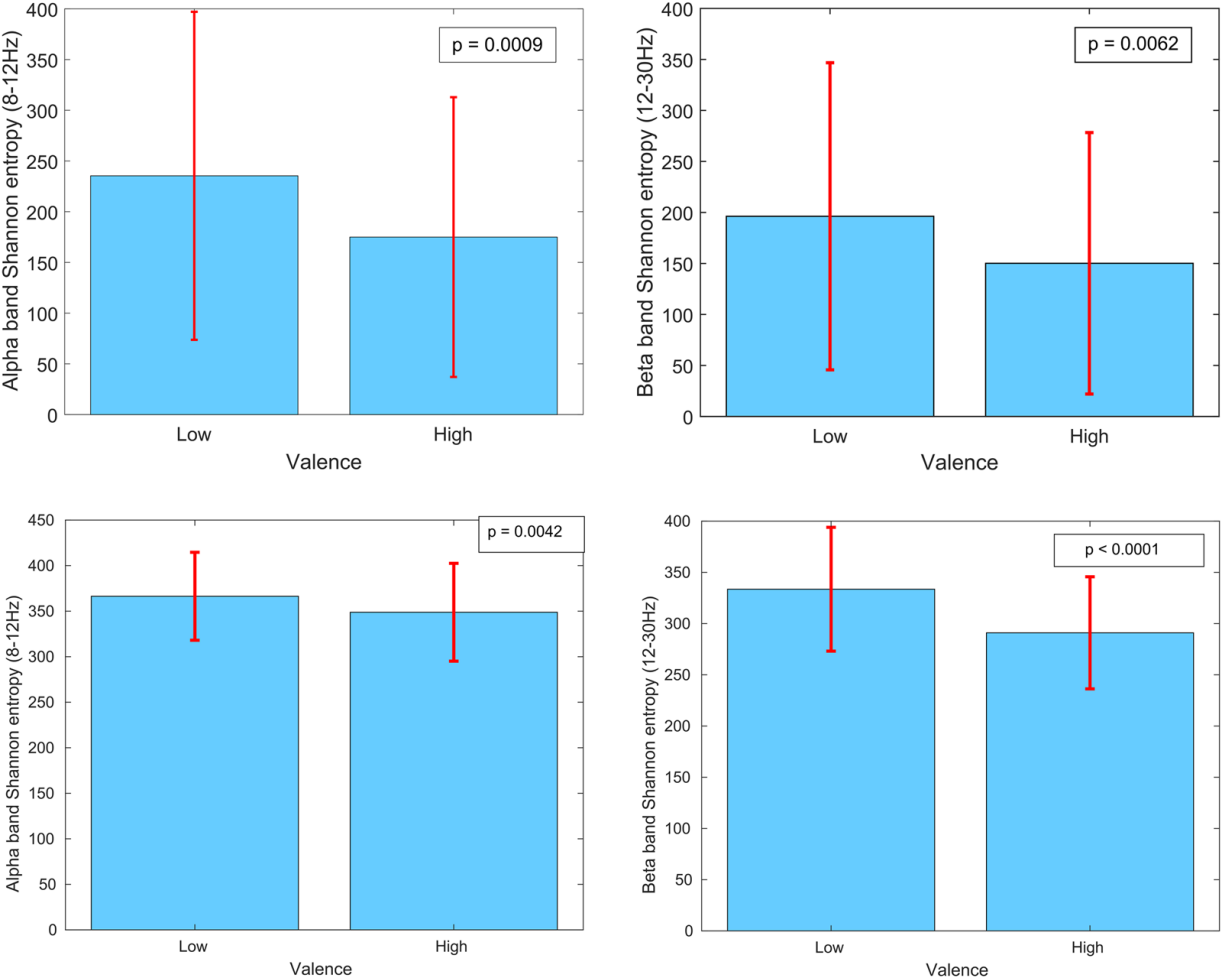
Mean and 95% confidence intervals of EEG asymmetry (left minus right) dynamics (as measured by Shannon entropy, measured in bits) in the alpha and beta frequency bands from all participants, while listening to the generated music (top row) and classical music (bottom row).

We also explored the dynamics of EEG asymmetry in the alpha and beta bands (the bands in which significant differences in asymmetry were observed) by inspecting the Shannon entropy of the EEG in trials during which participants reported high and low valence. A significant difference was found in entropy between these groups of trials in both frequency bands (p < 0.05, corrected for multiple comparisons via Holm-Bonferroni correction). Figure 7 illustrates this difference occurs during both the generated music listening and classical music listening trials.

Larger Shannon entropy was observed for trials in which participants reported lower valence. This indicates greater disorder in the EEG asymmetry during these trials. Thus, we can conclude that for trials in which the music-induced valence is lower, EEG is lower in the left prefrontal cortex and more disordered.

### EEG informed fMRI

We next sought to explore the relationship between activity in the sub-cortical emotion networks and the pre-frontal EEG asymmetry.

A time series of EEG asymmetry was first constructed via the method described above. This was then used as an independent variable to construct a regression model to analyse fMRI over all participants. Voxels found to co-vary significantly with the EEG measure of asymmetry indicate those regions of the brain which contain BOLD activity that is significantly related to pre-frontal asymmetry changes in EEG during the music-induced changes in affect

**Figure 8:**
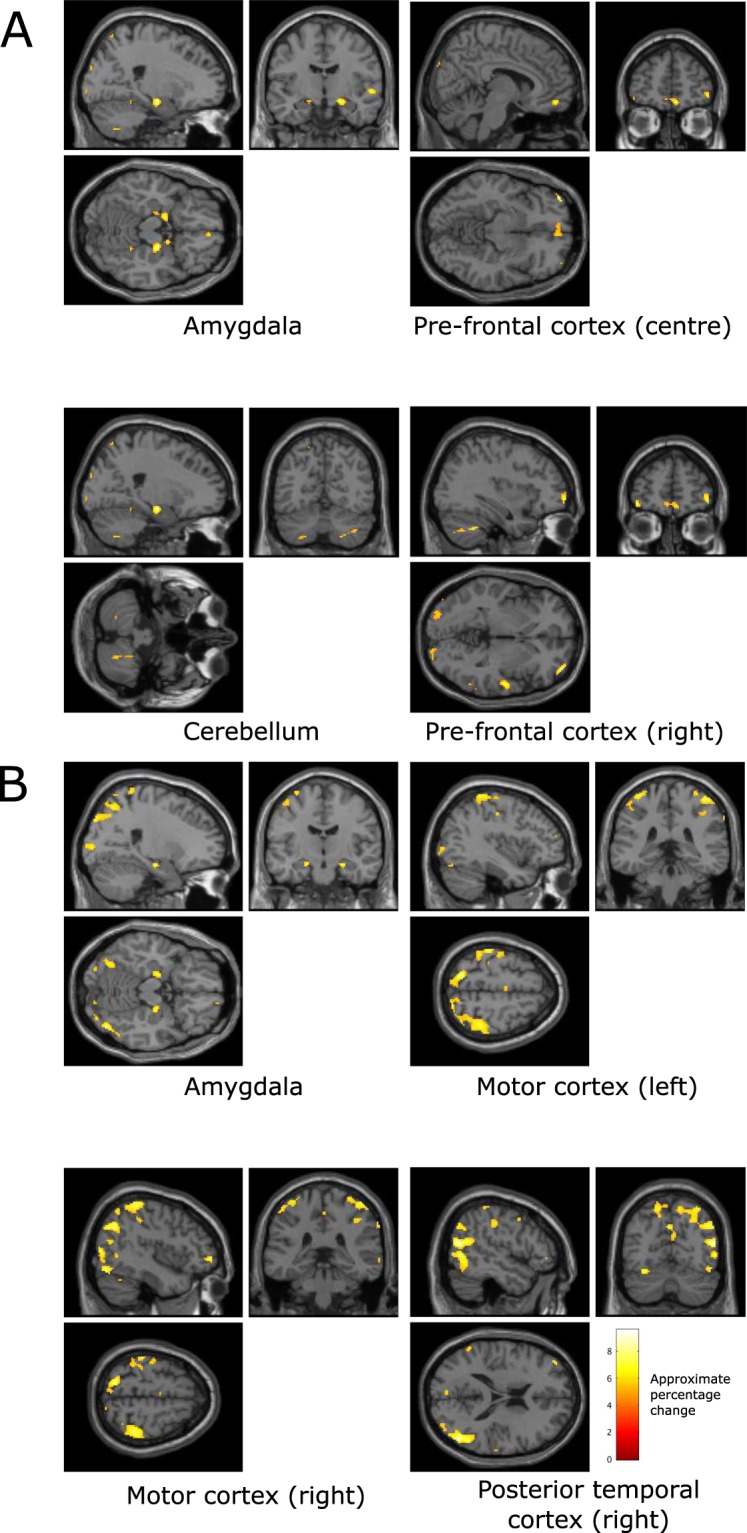
Brain regions that were found, over all participants, to co-vary significantly with measures of pre-frontal asymmetry recorded from the alpha band (**A**) and from the beta band (**B**) during the generated music listening tasks (p < 0.01, family wise error rate corrected). The colour scale indicates the approximate percentage change in BOLD activity in voxels where significant change occurs between conditions and is defined via SPM.

The clusters of the voxels that were found to co-vary significantly with EEG pre-frontal asymmetry during the generated music listening tasks (shown in Fig. 8) include regions in the amygdala, prefrontal cortex, cerebellum, motor cortex, and posterior temporal cortex (see Table 4). Voxel clusters that significantly co-vary with EEG asymmetry during classical music listening are similar and also include the prefrontal cortex and the occipital cortex (see Table 5).

**Table 4:** Local maxima of regions with significant co-variation with measured alpha and beta asymmetry, but not with movement, during generated music listening tasks (voxel size: 2 × 2 × 2 mm).

Anatomical location	Frequency band	Voxels	MNI coordinates (mm)	p (FWE corrected, voxel level)	F score
Amygdala, right hemisphere	Alpha	87	22, −12, −14	<0.001	6.40
Amygdala, left hemisphere	Alpha	52	−14, −2, −14	<0.001	7.63
Cerebellum	Alpha	139	36, −48, −40	<0.001	7.19
Amygdala, right hemisphere	Beta	30	24, −14, −18	<0.001	4.57
Amygdala, left hemisphere	Beta	40	−18, −10, −16	<0.001	6.71
Motor cortex, left hemisphere	Beta	737	−34, −42, 68	<0.001	7.59
Motor cortex, right hemisphere	Beta	1697	20, −88, 40	<0.001	7.63
Posterior temporal cortex	Beta	1169	52, −66, 16	<0.001	6.41

**Table 5:** Local maxima of regions with significant co-variation with measured asymmetry, but not with movement, during classical music listening tasks (voxel size: 2 × 2 × 2 mm).

Anatomical location	Frequency band	Voxels	MNI coordinates (mm)	p (FWE corrected, voxel level)	F score
Posterior cortex	Alpha	5965	−2, −58 56	0.001	7.66
Pre-frontal cortex	Alpha	205	−4, 58, −6	<0.001	6.41
Cerebellum	Alpha	238	−42, −42, −26	<0.001	18.01
Occipital cortex	Alpha	968	−18, −100, −8	<0.001	8.61
Cerebellum	Beta	248	−40, −44, −24	<0.001	16.61
Posterior cortex	Beta	2208	−12, −54, 68	<0.001	6.45
Amygdala, right hemisphere	Beta	13	26, −18, −14	<0.001	5.93

#### EEG asymmetry entropy informed fMRI

**Figure 9:**
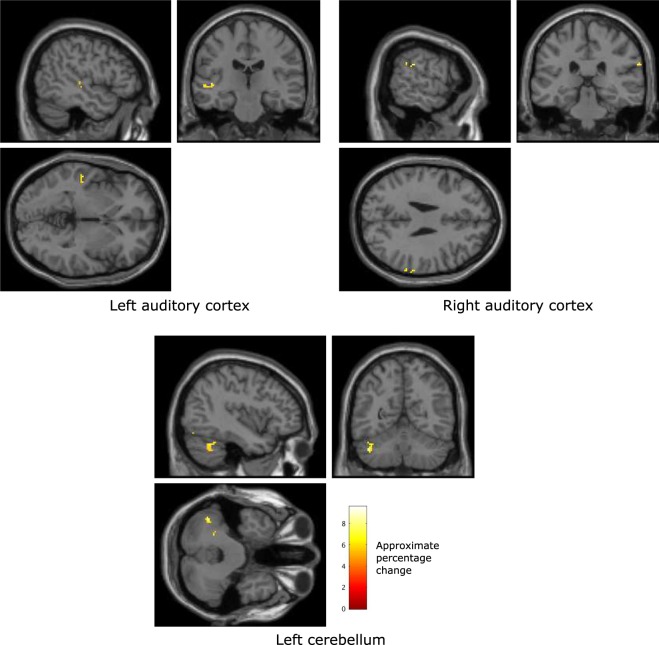
Brain regions that were found, over all participants, to co-vary significantly with measures of pre-frontal beta asymmetry dynamics, measured by Shannon entropy, recorded during the generated music listening tasks (p < 0.01, family wise error rate corrected). The colour scale indicates the approximate percentage change in BOLD activity in voxels where significant change occurs between conditions and is defined via SPM.

We also explored the relationship between EEG asymmetry entropy and BOLD throughout the brain. The regions of the brain that were observed to co-vary significantly with the Shannon entropy measure of the prefrontal EEG asymmetry dynamics, but were not related to use of the FEELTRACE interface, are illustrated in Fig. 9.

The locations, sizes, and significance of the cluster maxima’s that were found to co-vary significantly with the entropy of the EEG asymmetry during the generated music listening tasks include BOLD activity in the left and right auditory cortices (see Table 6). Additionally, activity in the left of the cerebellum significantly co-varied with asymmetry entropy. This may be contrasted with the regions observed to co-vary with asymmetry, which do not include the auditory cortices.

**Table 6:** Local maxima of regions with significant co-variation with measured asymmetry dynamics (measured by Shannon entropy), but not with movement, during the generated music listening tasks (voxel size: 2 × 2 × 2 mm).

Anatomical location	Voxels	MNI coordinates (mm)	p (FWE corrected, voxel level)	F score
Auditory cortex, left hemisphere	19	−54, −20, −2	<0.001	4.36
Auditory cortex, right hemisphere	5	36, 60, −6	<0.001	4.10
Cerebellum	198	−4, −78, −38	<0.001	4.91
Occipital cortex	144	0, −62, 68	<0.001	4.60

## Discussion

Music can have a profound effect on emotion and involves a large network of both cortical and sub-cortical brain regions^3^. Previous studies have identified a network of subcortical brain regions that reflect music-induced changes in emotion, including the limbic and paralimbic systems^6,8,71^. It has been suggested that the ability of music to induce changes in neural activity in these brain regions shows that it has potential as a therapeutic tool for a large number of diseases and conditions^3,11^. At the same time, separate EEG-based studies have shown that activity in the prefrontal cortex exhibits changes in asymmetry in response to changes in music-induced emotion^26–28^. However, so far we do not have a detailed understanding of the functional relationship between these disparate processes.

Our results show that, when listening to music, BOLD activity recorded from voxels in regions related to processing sound and speech (specifically, the auditory cortex^72^ and the posterior temporal cortex^73^) significantly co-vary with reports of felt valence. Our results also show that prefrontal EEG asymmetry changes in response to music-induced emotions reflect activity in several sub-cortical brain regions. Specifically, the posterior temporal cortex, amygdala, and cerebellum all exhibit significant correlations with affect and changes in prefrontal asymmetry in the EEG.

These sub-cortical brain regions form a part of both the limbic and paralimbic systems^3,6^, core parts of the emotion response network. Thus, our results highlight that prefrontal asymmetry in the EEG provides a reliable neural indicator of changes in activity in these systems as a result of changes in music-induced affect.

It is interesting to note that the auditory cortex activity (observed to co-vary with affect) does not significantly co-vary with EEG asymmetry. The reasons for this are not immediately clear, but it may be that only activity within the core emotion networks are reflected in EEG asymmetry, while brain regions that underlie autonomic and muscular responses to music^3^ are not reflected in changes in EEG asymmetry.

Furthermore, the sub-cortical brain regions that significantly co-vary with EEG asymmetry during classical music listening are found to be similar to those observed during generated music listening (the amygdala, cerebellum, and posterior temporal cortex), suggesting a common network of regions relates asymmetry to valence across different music types.

Changes in an individual’s affective state have been widely reported to elicit changes in pre-frontal asymmetry in the EEG^24,74^. These changes have been observed in a range of different frequency bands including the delta (0–4 Hz), theta (4–8 Hz), alpha (8–13 Hz), and beta (13–25 Hz) bands^21,75^. Our previous research suggests that the use of a Laplacian re-referencing scheme in the beta frequency band (13–30 Hz) produces a clear difference in asymmetry values that differentiates high and low felt valence conditions while participants listen to music^66^.

Our results show that EEG asymmetry in the alpha and beta bands is significantly larger when participants report high valence compared to when they report low valence. This corresponds to greater left hemisphere activity when participants report experiencing positively valenced emotions, which is supported by results reported elsewhere^23,24,75,76^.

Some prior work (e.g. work by Kenynan and colleagues^41^) has shown that the EEG can predict activity in the amygdala for some non-music-induced changes in affective state. Our results identify a larger set of brain regions in the emotion response network that reflect activity in the EEG. Importantly, we show they do so for music, an ecologically relevant, socio-affective stimulus^8^ known to involve some sub-cortical brain regions unique from other affective stimuli^3^.

Interestingly, we found that changes in EEG prefrontal asymmetry did not reflect changes in activity in the brain regions that underlie autonomic and muscular responses to music, such as the auditory cortex^3^. This suggests that, in the case of music-induced changes in valence, EEG prefrontal asymmetry just reflects affective responses to music.

Our results show that both generated music and classical music reveal BOLD activity in parts of the limbic system, including the amygdala, auditory cortex, cerebellum, and cingulate gyrus, that co-varies with reports of felt valence during music listening.

The amygdala is a key component of the emotion response network and has been widely reported to be involved in emotional processing^5,17,77,78^. Consistent with this, we found that activity in this region of the brain co-varied with music-induced changes in valence.

The auditory cortex in both the right and left hemispheres was observed to exhibit BOLD activity that co-varied with changes in valence, but not with EEG asymmetry. This brain region is involved in music appreciation, speech recognition, and a large number of other acoustic tasks^72^. It has also been observed to be involved in music-induced emotions^79^ but is not a part of the limbic or paralimbic systems and has been suggested to be more involved in autonomic and muscular responses to music than with affective responses to music^3^. Our results show that, although activity in this brain region is related to changes in valence, its activity is not reflected in EEG asymmetry, suggesting that it is not a core part of the emotion response network in the case of music listening.

The cerebellum is also known to be part of the network of regions that are involved in processing emotions^80^ and the cingulate gyrus has also been reported to relate to changes in music-induced emotions^81^. Thus, our results provide further evidence for the involvement of these regions in music-induced valence. However, there is only a small amount of evidence to suggest that all these brain regions respond together to changes in music-induced valence and that these effects are consistent over different pieces of music and multiple styles and genres of music^3^.

**Figure 10:**
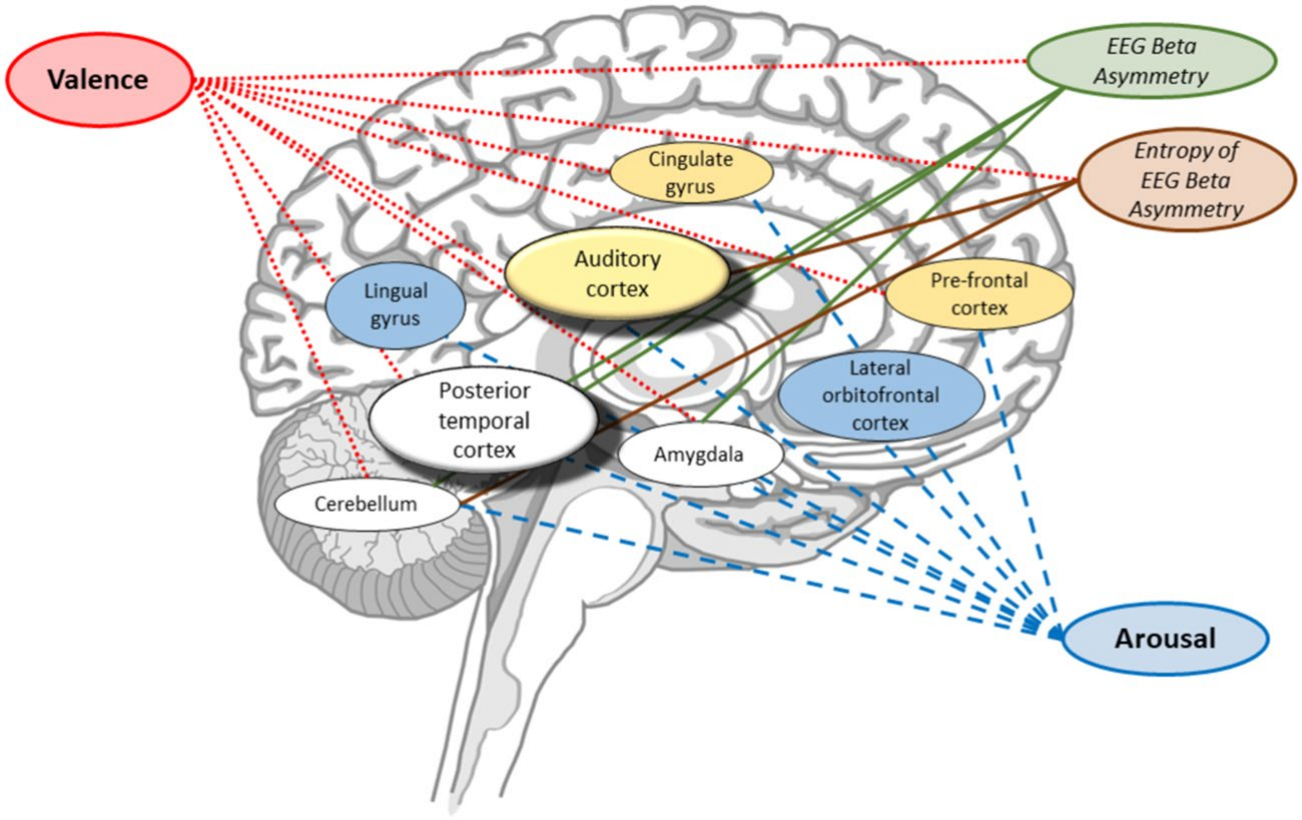
Schematic of brain regions found to exhibit significant changes in BOLD activity in relation to music-induced changes in valence, arousal, and/or changes in prefrontal asymmetry and the entropy of that asymmetry recorded via the EEG. Colours indicate observed significant associations between measured effects (valence, arousal, beta asymmetry) and changes in BOLD signal. Specifically, blue indicates that a region’s BOLD activity relates to reported arousal only, yellow indicates that a region’s BOLD activity relates to both valence and arousal, and white indicates that a region’s BOLD activity relates to valence, arousal, and measured EEG beta band asymmetry. The line colours and textures (solid or dashed) indicate significant relationships between brain regions and measured processes. Specifically, red dotted lines indicate which brain regions exhibit significant changes with reported valence, green solid lines indicate regions that significantly change with EEG asymmetry in the beta band, red solid lines indicate regions that significantly change with the entropy of the EEG asymmetry, and blue dotted lines indicate regions that significantly change with reported arousal. Note that the brain regions are positioned approximately at their relative locations in the brain, but positioning is not intended to be anatomically precise.

Our results suggest a set of functional relationships between the sub-cortical emotion response network and EEG asymmetry, as illustrated in Fig. 10. Regions are highlighted and links to associated effects (valence, arousal, and EEG asymmetry) are illustrated.

Thus, our results provide evidence that EEG can be used as a neural correlate of changes in activity in the limbic and paralimbic systems in response to music.

Specifically, analysis of EEG responses to music-induced changes in emotion reveals significant concomitant variation in asymmetry within the alpha and beta bands. This matches our earlier observations that beta prefrontal asymmetry relates to changes in valence^66^.

Subsequent EEG-informed fMRI analysis identified brain regions that co-vary with changes in alpha and beta asymmetry. This revealed a collection of regions, including the amygdala, the posterior temporal cortex, and the cerebellum, to be related to changes in asymmetry in both frequency bands.

This is the first such result revealing the relationship between deep brain regions and changes in cortical EEG asymmetry related to modulation of music-induced valence. It reveals that these three key parts of the emotion response network relate to, and can be predicted by, measures of EEG asymmetry, while other parts of the emotion response network that relate to reports of music-induced valence (such as the auditory cortex and the cingulate gyrus) are not predicted by the alpha or beta asymmetry measures.

A number of brain regions exhibit significant correlations between BOLD activity, EEG asymmetry, and participant reports of affect. Specifically, the posterior temporal cortex, amygdala, and cerebellum all exhibit significant correlations with affect and EEG. This highlights the appropriateness of EEG asymmetry as a measure of music-induced changes in activity in the emotion response network.

Our results show that there is a significant difference in the dynamics of the prefrontal asymmetry between high and low valence during music listening. Previous studies have shown that measures of Shannon entropy of the wavelet decomposition of EEG can be used to classify high vs low valence^44,45,68^, and our results provide further explanation for the mechanism behind this result. Specifically, our results indicate that prefrontal asymmetry becomes more disordered during music-induced decreases in valence.

Changes in the dynamics of the prefrontal asymmetry were also observed to relate to changes in BOLD within the auditory cortices in both the left and right hemispheres as well as the cerebellum. The activity in all three of these areas was also observed to relate to valence and this suggests these areas act, as a part of the affective response network, to modulate the temporal dynamics of the prefrontal EEG asymmetry.

It is also interesting to note that distinct sub-cortical regions significantly co-vary with asymmetry and the Shannon entropy of that asymmetry. For example, BOLD activity in the auditory cortices co-varies with entropy of the asymmetry but not directly with asymmetry. The auditory cortex is involved in acoustic processing and in autonomic and motor responses to music^3^. Thus, it may be that the entropy of the EEG asymmetry reflects autonomic responses to music, while magnitude of the asymmetry reflects affective responses to music.

Changes in music-induced arousal and changes in valence were observed to involve similar sets of brain regions. Specifically, the amygdala, posterior temporal cortex, cerebellum, cingulate gyrus, auditory cortex, and pre-frontal cortex were observed to exhibit concomitant changes in BOLD with changes in arousal. These results are partly in-line with those of Heller (1993) and Rogenmoser (2016), who both report involvement of the right posterior temporal cortex in affect^27,28^.

In addition, the lingual gyrus and lateral orbitofrontal gyrus both exhibited BOLD activity that co-varied with arousal. This suggests that processing changes in arousal recruits more brain regions than processing changes in valence. Furthermore, it highlights that the axes of the valence-arousal model of affect^14^ involve distinct, yet inter-related, cortical networks.

The lingual gyrus has been reported to be involved in affective responses to emotional faces^82^. It has also previously been reported to exhibit activity that changes with the induced affect of video clips^83^ and changes in picture-induced arousal^84^.

The orbitofrontal gyrus has been reported to be involved in response inhibition^85^ and the processing of vocally conveyed emotions and making emotional judgments^86^. The orbitofrontal cortex is also a part of the brain network that underlies autonomic responses to music^3^. Thus, our finding that the orbitofrontal cortex co-varies with arousal, but not valence, is somewhat surprising. One possible explanation is that less tonal music is more challenging for listeners, which could make them more alert and increase arousal^87^. It should be highlighted that our music stimulus choices were not deliberately atonal, but that perceived tonality of music is a continuously varying process^88^.

It may be noted that the prefrontal cortex was also observed to exhibit changes in BOLD during changes in valence, but only while participants listened to classical music, not generated music. This could be due to the larger affective response induced by classical music, which is more complex and varied than our generated music.

Additionally, unlike the generated music, some of the classical music is likely to have been familiar to the participants. Thus, effects of memory are more likely to affect participants’ responses to the classical music^1^, potentially resulting in larger inter-participant differences.

The two types of music essentially serve as controls to one another in our study. Generated music is used to avoid effects of familiarity and repeated listening that can arise from the use of well-known pieces of music^48^, while the classical music is used to induce changes in affect that may not be possible with synthetic music. Thus, changes in activity in the emotion response network that arise from both stimuli types are much more likely to be a genuine result of changes in affect. It is outside the scope of this work to investigate contrasts between these two stimuli types here.

It is interesting to note that, although we identified several brain regions that have previously been reported to be involved in affective responses to music, we did not identify all the regions that are commonly reported to relate to affect. Specifically, we did not observe changes in BOLD in the thalamus or hypothalamus.

Both the thalamus and hypothalamus have been reported to be involved in affective responses to music^18^. However, it is possible that the effects evoked by our music are not sufficient to produce significant effects in these brain regions.

Additionally, we did not observe significant relationships between EEG in the theta frequency band and sub-cortical BOLD activity. This is somewhat surprising, as EEG activity in the theta band has been linked to music-induced changes in valence in a number of studies^26,28,32,66^. However, in some of these studies the EEG theta band activity was measured over the midline (running across the centre of the brain from front to back), and this is linked to changes in music-induced valence. Our present study only looked at the prefrontal asymmetry in the theta band and it is possible that our prefrontal theta asymmetry measure is not related to BOLD fMRI activity with sufficient power for us to observe it clearly.

A set of co-variations in brain activity have previously been reported between sub-cortical regions during changes in affective states. These include connections between the amygdala and the hypothalamus, while the hypothalamus has also been widely reported to share connections with a number of regions during affect, including the cingulate gyrus and the insula^89^. Our findings add additional information to some of these reports (in particular, the concomitant activation of the amygdala and several other key brain regions further demonstrates the presence of co-variations between brain activity and affective responses), while adding other regions to this map.

When considering our results, it is important to keep some caveats in mind. First, the conservative significance testing used in our analysis (family-wise error correction, p < 0.01) means some regions that exhibit weak, yet meaningful, relationships with affective responses to music might not be identified as significant.

Second, we used a control condition in which participants were required to use the FEELTRACE interface to perform the same movements (both arm movements and associated eye movements) that they made during the music listening and reporting trials. This allowed us to identify brain regions related to the use of FEELTRACE and discount them when considering which brain regions were involved in music-induced changes in affect.

However, this approach makes the explicit assumption that the brain regions involved in music-induced changes in affect are distinct from regions involved in the control of FEELTRACE, an assumption that may not always be valid. For example, the motor cortex has been reported to exhibit activity entrained to music tempo^90^ and is also involved in coordination of hand movements to control FEELTRACE.

Each piece of music was played twice to each participant, once with FEELTRACE and once without. This was intended to mitigate, in part, the effect of excluding regions that are involved strongly in affect and control of FEELTRACE, as discussed above. Specifically, a region that is strongly involved in affective responses but also weakly involved in control of FEELTRACE will be found to co-vary significantly with participant reports of their affect in twice as many trials as the FEELTRACE reporting only trials, reducing its probability of exclusion.

However, to achieve this mitigation of the effect of control of FEELTRACE, participants are assumed to respond to the same stimuli the same way during ‘music only trials’ and ‘music and reporting trials’ (the reports from the ‘music and reporting trials’ are copied to the ‘music only trials’). It may be argued that this assumption does not hold, as repeated listening changes a listener’s response^48^. However, this is mitigated by random ordering and timing of the trials.

Of additional interest, the right primary motor cortex is involved in changes in valence. All participants controlled FEELTRACE with their right hand, which would be expected to involve the left primary motor cortex^91^. Additionally, movement related BOLD activity (along with the activity in the visual cortex) was controlled for. Thus, this activation is highly likely to be related to processing the valence of the music.

One possible explanation is that changing valence relates to tempo and predictability of the music^3,92^. Both of these effects have been reported to relate to motor cortex activity^4,90,93^.

## Conclusions

Our study identifies, for the first time, how activity in the sub-cortical emotion response network concomitantly changes with changes in EEG asymmetry amplitudes and temporal dynamics. This includes differences in sub-cortical regions that co-vary with EEG asymmetry and the entropy of the asymmetry. Thus, the results support our first hypothesis, that prefrontal EEG asymmetry does significantly relate to activity in the sub-cortical emotion response network.

We also show that EEG during music-listening tasks can be used to predict activity in sub-cortical brain regions including the amygdala, posterior temporal cortex, and cerebellum. Thus, the results also support our second hypothesis, that EEG correlates with changes of activity within the limbic system.

## Data Availability

The data generated during this study are available from the corresponding author on reasonable request. Please note, availability of some data is subject to ethics and data protection restrictions, e.g. data that could be used to identify human participants will not be made available.
